# O03 Phenotypic antimicrobial resistance to last-resort antibiotics in carbapenemase-producing bacteria from Ukrainian patients

**DOI:** 10.1093/jacamr/dlad143.003

**Published:** 2024-01-03

**Authors:** Nelianne J Verkaik, Cornelia C H Wielders, Hans den Boer, Diana Langerak, Marius Vogel, Sandra Witteveen, Angela de Haan, Jeroen Bos, Mireille van Westreenen, Daan W Notermans, Antoni P A Hendrickx

**Affiliations:** Department of Medical Microbiology and Infectious Diseases, Erasmus University Medical Center, Rotterdam, The Netherlands; Centre for Infectious Disease Control (CIb), National Institute for Public Health and the Environment (RIVM), Bilthoven, The Netherlands; Department of Medical Microbiology and Infectious Diseases, Erasmus University Medical Center, Rotterdam, The Netherlands; Department of Medical Microbiology and Infectious Diseases, Erasmus University Medical Center, Rotterdam, The Netherlands; Department of Medical Microbiology and Infectious Diseases, Erasmus University Medical Center, Rotterdam, The Netherlands; Centre for Infectious Disease Control (CIb), National Institute for Public Health and the Environment (RIVM), Bilthoven, The Netherlands; Centre for Infectious Disease Control (CIb), National Institute for Public Health and the Environment (RIVM), Bilthoven, The Netherlands; Centre for Infectious Disease Control (CIb), National Institute for Public Health and the Environment (RIVM), Bilthoven, The Netherlands; Department of Medical Microbiology and Infectious Diseases, Erasmus University Medical Center, Rotterdam, The Netherlands; Centre for Infectious Disease Control (CIb), National Institute for Public Health and the Environment (RIVM), Bilthoven, The Netherlands; Centre for Infectious Disease Control (CIb), National Institute for Public Health and the Environment (RIVM), Bilthoven, The Netherlands

## Abstract

**Background:**

Since March 2022, there has been an emergence of MDR microorganisms (MDRO) in Europe because of an increasing number of Ukrainian patients with MDRO. The goal was to collect large-scale phenotypic susceptibility data to assess implications for clinical practice.

**Methods:**

For 122 MDRO (carbapenemase-producing Enterobacterales (CPE, *n*=96), *Pseudomonas aeruginosa* CPPA (*n*=20), carbapenem-resistant *Acinetobacter baumannii*-*calcoaceticus* (CRAB, *n*=6), collected from March to December 2022, broth microdilution (BMD), fosfomycin agar dilution, gradient strip ampicillin/sulbactam, sulbactam/durlobactam, ceftazidime-avibactam-aztreonam and disc diffusion (DD) cefiderocol was performed. Epidemiological data was obtained as part of mandatory reporting by Municipal Health Services (CPE), and surveillance questionnaires (CPPA/CRAB).

**Results:**

Admission at a Ukrainian hospital in the last year was a risk factor for acquiring MDRO. Of all isolates, 65% (79/122) were *bla*_NDM_-positive. For meropenem, aminoglycosides, ceftazidime-avibactam, ceftolozane-tazobactam and imipenem-relebactam susceptibility rates were low (0-30%). For cefiderocol, results depended on reading with or without microcolonies, applying EUCAST or CLSI breakpoints, and whether DD or BMD was used: e.g. for *Klebsiella pneumoniae*, 30%–97% were susceptible, depending on these variables. For colistin, 103/111 (93%) non-intrinsically resistant CPE/CPPA/CRAB isolates were susceptible. For the majority of CPE, a low MIC of <0.5 mg/L was measured for tigecycline and ceftazidime-avibactam-aztreonam. For CPPA, cefiderocol tested susceptible in 65%–100% of isolates. For CRAB, ampicillin/sulbactam MICs were ≥128 mg/L, for sulbactam/durlobactam 1–2 mg/L.

**Conclusions:**

There is extensive phenotypic resistance to last-resort antibiotics in carbapenemase-producing bacteria from Ukrainian patients. For cefiderocol, interpretation of susceptibility results depends on a number of variables. In case of infection, treatment options are colistin, cefiderocol (CPE/CPPA/CRAB), tigecycline (CPE/CRAB), ceftazidime-avibactam-aztreonam (CPE), sulbactam/durlobactam (CRAB).

